# Supplementary material for: Investigation of growth curves with different nonlinear models and MARS algorithm in broiler chickens
Source: PLoS One. 2024 Nov 22;19(11):e0307037. doi: 10.1371/journal.pone.0307037 (PMC11584083; doi:10.1371/journal.pone.0307037)
Supplement: S1 Table — (DOCX) [file pone.0307037.s001.docx]

**S1 Table**

**Table 1.** **Growth model parameters for live weight in broiler chickens.**

| **Parameters** | **Logistics** | **Gompertz** | **Weibull** | **Hossfeld** | **Von Bertalanffy** |
| --- | --- | --- | --- | --- | --- |
| A | 3136.722 | 6854.354 | 13500.860 | 8872.315 | 20692.727 |
| B | 37.106 | 4.882 | 13436.664 | - | 0.885 |
| K | 0.736 | 0.241 | 0.005 | - | 0.086 |
| Λ | - | - | 1.941 | - | - |
| b1 | - | - | - | 106.170 | - |
| b2 | - | - | - | 1.963 | - |

**Table 2.** **Goodness of fit statistics for different non-linear growth models in chicken.**

| **Statistics** | **Logistics** | **Gompertz** | **Weibull** | **Hossfeld** | **Von Bertalanffy** |
| --- | --- | --- | --- | --- | --- |
| R^2^ | 0.9997 | 0.9998 | 0.9996 | 0.9960 | 0.9996 |
| Adj. R^2^ | 0.9995 | 0.9997 | 0.9993 | 0.9940 | 0.9994 |
| MSE | 629.327 | 470.570 | 1085.258 | 3182.381 | 821.485 |
| RMSE | 25.086 | 21.681 | 32.943 | 56.413 | 28.662 |
| AIC | 70.357 | 68.750 | 74.723 | 78.934 | 72.317 |
| BIC | 70.802 | 68.934 | 75.117 | 79.227 | 72.829 |
| IPT | 4.912 | 6.579 | 10.556 | 6.074 | 11.354 |
| IPW | 1568.361 | 2521.578 | 6851.335 | 2176.271 | 6130.963 |
| Final body weight | 2164.56 | 2176.04 | 2177.50 | 2138.51 | 2175.33 |

IPT: Point of inflection time, IPW: Point of inflection weight.

**Table 3. Observed average body weights (g) and estimated body weights by different methods.**

| **Weeks** | **Observed weight** | **Logistics** | **Gompertz** | **Weibull** | **Hossfeld** | **Bertalanffy** | **MARS** |
| --- | --- | --- | --- | --- | --- | --- | --- |
| 0 | 47.68 | 82.32 | 51.95 | 64.20 | 27.19 | 31.79 | 46.73 |
| 1 | 151.76 | 167.02 | 148.01 | 134.94 | 82.79 | 138.11 | 140.07 |
| 2 | 352.37 | 329.50 | 336.88 | 333.90 | 314.36 | 343.78 | 364.06 |
| 3 | 613.21 | 617.18 | 642.79 | 649.68 | 668.09 | 656.31 | 588.05 |
| 4 | 1033.27 | 1061.10 | 1067.8 | 1070.93 | 1111.73 | 1072.64 | 1021.00 |
| 5 | 1607.70 | 1619.11 | 1590.9 | 1584.89 | 1611.92 | 1583.31 | 1586.73 |
| 6 | 2193.10 | 2164.56 | 2176.04 | 2177.50 | 2138.51 | 2175.33 | 2191.96 |
