# Supplementary material for: Investigation of growth curves with different nonlinear models and MARS algorithm in broiler chickens
Source: PLoS One. 2024 Nov 22;19(11):e0307037. doi: 10.1371/journal.pone.0307037 (PMC11584083; doi:10.1371/journal.pone.0307037)
Supplement: Attachment — Submitted filename: RESPONSE LETTER-PONE.docx [file pone.0307037.s002.docx]

**RESPONSE LETTER**

This document is a response letter on the manuscript ***#*** **PONE-D-24-10742 *entitled"*** **Investigation of growth curves with different nonlinear models and MARS algorithm in broiler chickens*".***

Dear Editor,

Many thanks for sharing valuable comments of you and reviewers with us on improving the manuscript # PONE-D-24-10742 entitled "Investigation of growth curves with different nonlinear models and MARS algorithm in broiler chickens". We are happy that the manuscript will be acceptable for evaluation in "Plos One". We have given answers to all comments of the reviewers evaluating our manuscript using red, green and blue color fonts. Also, red (Reviewer 1), green (Reviewer 2), and blue (Editor) color font on the revised manuscript has been used for indicating all the corrections made by Reviewers.

With Best Regards

Assoc. Prof. Şenol Çelik

Corresponding author

**COMMENTS FOR THE AUTHOR**

**Reviewer 1**

**Recommend**

1. Graphical abstract to be add. it gives additional understanding of the process flow of the work

***Answer***

*"The actual measured live weight values and the weight values estimated by Logistics, Gompertz, Weibull, Hossfeld, Von Bertalanffy models and MARS algorithm are close and in harmony with each other in the graph. However, the weight values estimated from the MARS algorithm are much closer to the observed live weight values", the statement added.*

**Recommend**

2. Modeling section to be improve with all the parameter expansion in the text

***Answer***

*Modeling section was improved with all the parameter expansion in the text.*

**Recommend**

3. Author advised to refer the latest article for citation to get current trends/gab on this field work

***Answer***

*Both in the introduction and in the discussion section, current studies were included.*

*Done (Line 55-74 and Line 355-377).*

**Recommend**

4. Provide the justifications how all the proposed approaches given approximately same level of outcomes. If possible use other representations to express the outcomes of the various methodology results.

***Answer***

Data checked and re-analysed. Some changes that occurred as a result of the analysis are marked in green. The results for all methodologies used were close to each other. However, goodness-of-fit tests showed that the Gompertz model gave better results than the others among the growth models. When the MARS algorithm was included, the MARS algorithm gave the best result when all models were compared. In addition, the graph of the growth models together is shown in Figure 3. In addition, the graph of the MARS algorithm and Gomperz model together, which gave the best prediction results with the observed values, is shown in Figure 4.

**Reviewer 2**

The authors have done a good research work by comparing different growth curve models. The authors are recommended to consider the comments.

**Recommend**

1. The ethical clearance details are given to carry out the chicken study.

***Answer***

*Done.*

*It is given in the Materials and Methods section. "Institutional review board statement: This is an individual study, hence no institutional review or ethics committee is necessary. The study did not include any persons".*

**Recommend**

2. Before starting the experiment, the breeding conditions of the animals were not clearly mentioned.

***Answer***

*Necessary information about the breeding and reproduction conditions of animals was written (Line 87-100).*

**Recommend**

Line 13-14: It would have been good to highlight the recommended solutions to current problems within "Horticultural Crop Secondary Metabolism".

***Answer***

Highlighted in the proposed solutions to existing problems within the scope of "Secondary Metabolism in Horticultural Plants" (Line 13-14).

**Recommend**

3. In table 3, the body weight is mentioned as 0.00 for hossfeld model. How can it be possible?

***Answer***

*The analysis was redone and the predicted first week body weight for the Hossfeld model is 27.19, not 0.00. The first week body weight was corrected as 27.19.*

**Recommend**

4. In table 2, regression values are mentioned as normal but the body weight data are not clear.

***Answer***

*Since the results changed after the data were re-analysed, the information in Table 2 has been rearranged. Final body weight information was added next to the regression values.*

**Recommend**

5. Based on the graphical representation, all the models end at the same endpoint. Based on this results, how can you conclude that MARS algorithm and gompertz are good?

***Answer***

*The results of goodness of fit tests were more decisive. As a result of the goodness-of-fit tests, the MARS algorithm and the Gompertz growth model gave better results. However, in order to make this better understandable in the graph, we have removed the graph of Logistics, Gompertz, Weibull, Hossfeld and Von Bertalanffy models together with the observed values from Figure 2 and added them separately in Figure 3. In addition, we have given the graph of the observed values together with the predicted values from the MARS algorithm and Gompertz growth model in Figure 4.*

*In addition, as stated by Mr Editor, some minor text overlapping with a previously published study, which can be accessed at ‘https://doi.org/10.1007/s11250-021-02700-8’, has been revised.*
